# Bandgap Engineering of an Aryl-Fused Tetrathianaphthalene for Visible-Blind Organic Field-Effect Transistors

**DOI:** 10.3389/fchem.2021.698246

**Published:** 2021-05-28

**Authors:** Lijuan Zhang, Xinzi Tian, Yantao Sun, Jiarong Yao, Shuyuan Yang, Zheyuan Liu, Zhen Ge, Hongtao Zhang, Yan Sun, Xiangfeng Shao, Rongjin Li, Wenping Hu

**Affiliations:** ^1^ Tianjin Key Laboratory of Molecular Optoelectronic Sciences, Department of Chemistry, School of Science, Tianjin University and Collaborative Innovation Center of Chemical Science and Engineering, Tianjin, China; ^2^ State Key Laboratory of Applied Organic Chemistry, Lanzhou University, Lanzhou, China; ^3^ College of Materials Science and Engineering, Fuzhou University, Fuzhou, China; ^4^ State Key Laboratory and Institute of Elemento-Organic Chemistry, the Centre of Nanoscale Science and Technology and Key Laboratory of Functional Polymer Materials, Renewable Energy Conversion and Storage Center (RECAST), College of Chemistry, Nankai University, Tianjin, China; ^5^ Joint School of National University of Singapore and Tianjin University, International Campus of Tianjin University, Fuzhou, China

**Keywords:** bandgap engineering, visible-blind, organic semiconductors, organic field-effect transistors, molecular configuration

## Abstract

Stability problem of organic semiconductors (OSCs) because of photoabsorption has become a major barrier to large scale applications in organic field-effect transistors (OFETs). It is imperative to design OSCs which are insensitive to visible and near-infrared (VNIR) light to obtain both environmental and operational stability. Herein, taking a 2,3,8,9-tetramethoxy [1,4]benzodithiino[2,3-b][1,4]benzodithiine (TTN2) as an example, we show that controlling molecular configuration is an effective strategy to tune the bandgaps of OSCs for visible-blind OFETs. TTN2 adopts an armchair-like configuration, which is different from the prevailing planar structure of common OSCs. Because of the large bandgap, TTN2 exhibits no photoabsorption in the VNIR region and OFETs based on TTN2 show high environmental stability. The devices worked well after being stored in ambient air, (i.e. in the presence of oxygen and water) and light for over two years. Moreover, the OFETs show no observable response to light irradiation from 405–1,020 nm, which is also favorable for high operational stability.

## Introduction

Organic semiconductors have attracted tremendous interest in recent days because of their tailorable optoelectronic properties by molecular design, as well as their potential low cost, large-area fabrication, and mechanical flexibility (Ji, et al., 2019; Wang, et al., 2018; Wu, et al., 2020). Organic field-effect transistors are basic components in a wide range of applications, such as active matrix organic light-emitting diode (AMOLED) pixel circuits, radio frequency identification (RFID) tags and flexible logic circuits (Ren, et al., 2018; Wang, et al., 2020; Duan, et al., 2020). Although great progress has been made in OFETs and mobilities over 10 cm^2^ V^−1^ s^−1^ have been achieved by many OSCs (Dong, et al., 2012; Wang, et al., 2018), the stability issues are much less investigated. From a practical point of view, electronical applications require environmental as well as operational stability of the devices. OSCs typically possess high photoabsorption coefficients in the visible and near-infrared range, which means that incident photons can be efficiently absorbed by films less than a micrometer in thickness (Tang et al., 2008b; Kang, et al., 2011; Wang, et al., 2012; Yao, et al., 2020). The high photoabsorption coefficients of OSCs are favorable for application such as organic photovoltaics (OPVs), but lead to serious stability problems in OFETs because both mobilities and threshold voltages of OFETs change after photoabsorption by the photoconduction and/or photogating effects (Baeg, et al., 2013; Min, et al., 2019, 2021). For applications such as thin film transistor (TFT) drive of AMOLED, variations in mobility and/or threshold voltage hinder the fabrication of large AMOLED displays with high uniformity (Ke et al., 2020). What’s worse, material degradation may occur after photoabsorption (Forrest Stephen, 2015). One typical example is pentacene, which is a benchmark OSC for OFETs. Pentacene thin film shows strong photoabsorption in the visible range, which causes photooxidation in presence of light and air and produces transannular endoperoxide and other complex reaction products (Maliakal, et al., 2004). The photooxidation not only destroys the delocalized π-bonding and impedes charge transport but also shifts the threshold voltage, resulting in permanent device failure (Komoda, et al., 2003; Qiu, et al., 2003; Nádaždy, et al., 2007). The stability problem of OSCs caused by photoabsorption has become a major barrier to large scale applications in OFETs. It is imperative to design OSCs which are insensitive to VNIR light with both environmental and operational stability.

Herein, we show that controlling molecular configuration is an effective strategy to tune the bandgaps of OSCs for stable OFETs. 2,3,8,9-tetramethoxy [1,4]benzodithiino[2,3-b][1,4]benzodithiine (TTN2), whose skeleton is an isomer of dibenzo-tetrathiafulvalene (DB-TTF) (Mizuno and Cava, 1978), is used as an example of this study (Sun, et al., 2016). DB-TTF adopts a planar structure and shows a narrow optical bandgap of 2.36 eV. Its photoabsorption in the VNIR range poses serious stability problems (Mas-Torrent, et al., 2006a). By changing the molecular configuration from a planar configuration of DB-TTF to an armchair configuration of TTN2, the bandgaps increased substantially from 2.36 to 3.02 eV, making TTN2 an OSC without photoabsorption in the VNIR region. As a result, visible-blind OFETs with high environmental and operational stability were obtained. Device performance was largely maintained after the OFETs being kept in ambient air and light for over two years. Moreover, the devices were insensitive to light irradiation of wavelengths from 405–1,020 nm.

## Experimental Method

### TTN2 Microcrystal Growth

We had reported the synthetic procedures of TTN2 previously (Sun, et al., 2016). To grow its crystals, TTN2 powder was dissolved in a mixed solution with a m-xylene: toluene: dichloromethane volume ratio of 2 : 1: 1 at a concentration of 0.5 mg ml^−1^. Glass weighing bottles (40 mm × 25 mm) were used as the containers to grow the microcrystals. The bottles were cleaned by sonification in ethanol for 30 min. SiO_2_ (300 nm)/Si substrates were successively cleaned by sonification in deionized water (DI), acetone, and isopropanol. The substrates were then treated with oxygen plasma at 80 W for 10 min followed by immediate modification with octadecyltrichlorosilane (OTS) by a vapor phase method. The OTS-modified SiO_2_/Si substrates were successively cleaned by sonification in chloroform, n-hexane and isopropanol. The cleaned OTS-modified SiO_2_/Si substrates were then placed flat at the bottom of the glass weighing bottles. 20 μL of as-prepared TTN2 solution was slowly dropped on the surfaces of the substrates. The bottles were placed in an oven and kept at 60°C for 2 h. After completer evaporation of the solvents, microcrystals of TTN2 were obtained.

### Device Fabrication and Mobility Calculation

Bottom-gate/top-contact OFETs were constructed with OTS-modified SiO_2_ (300 nm) as the gate dielectric layer. Source and drain electrodes were fabricated by stamping Au (80 nm) stripes on TTN2 microcrystals (Tang, et al., 2008a). Field-effect mobility (*μ*) in the saturation regime was calculated from the following equation: *I*_DS_ = (*W*/2*L*)*µ* *C*_i_ (*V*_GS_−*V*_TH_)^2^, where *I*_DS_ is the source-drain current, *μ* is the field-effect mobility, *V*_TH_ is the threshold voltage, *V*_GS_ is the applied gate voltage, *L* is the channel length, *W* is the channel width and the *C*_i_ is the specific capacitance (10 nF cm^−2^).

### Instrumentation

Optical and cross-polarized optical microscope (OM and POM) images were obtained with Nikon ECLIPSE Ci-POL polarized optical microscope. Tapping mode atomic force microscopy (AFM) images were measured using a Bruker Dimension Icon. X-ray diffraction (XRD) measurements were carried out in reflection mode at 45 kV and 200 mA with monochromatic Cu Kα radiation utilizing a Rigaku Smartlab diffractometer. Ultraviolet-visible and near-infrared (UV-Vis-NIR) absorption spectrum of TTN2 microcrystals was measured with a Agilent Technologies Cary Series UV-Vis-NIR Spectrophotometer. OFETs were characterized using a Keithley 4200 SCS in ambient environment at room temperature. The photo responses of the OFETs were measured with lasers with tunable power intensity. The laser power intensity was measured *in situ* with a PM100 digital power meter.

### Results and Discussion

**FIGURE 1:**
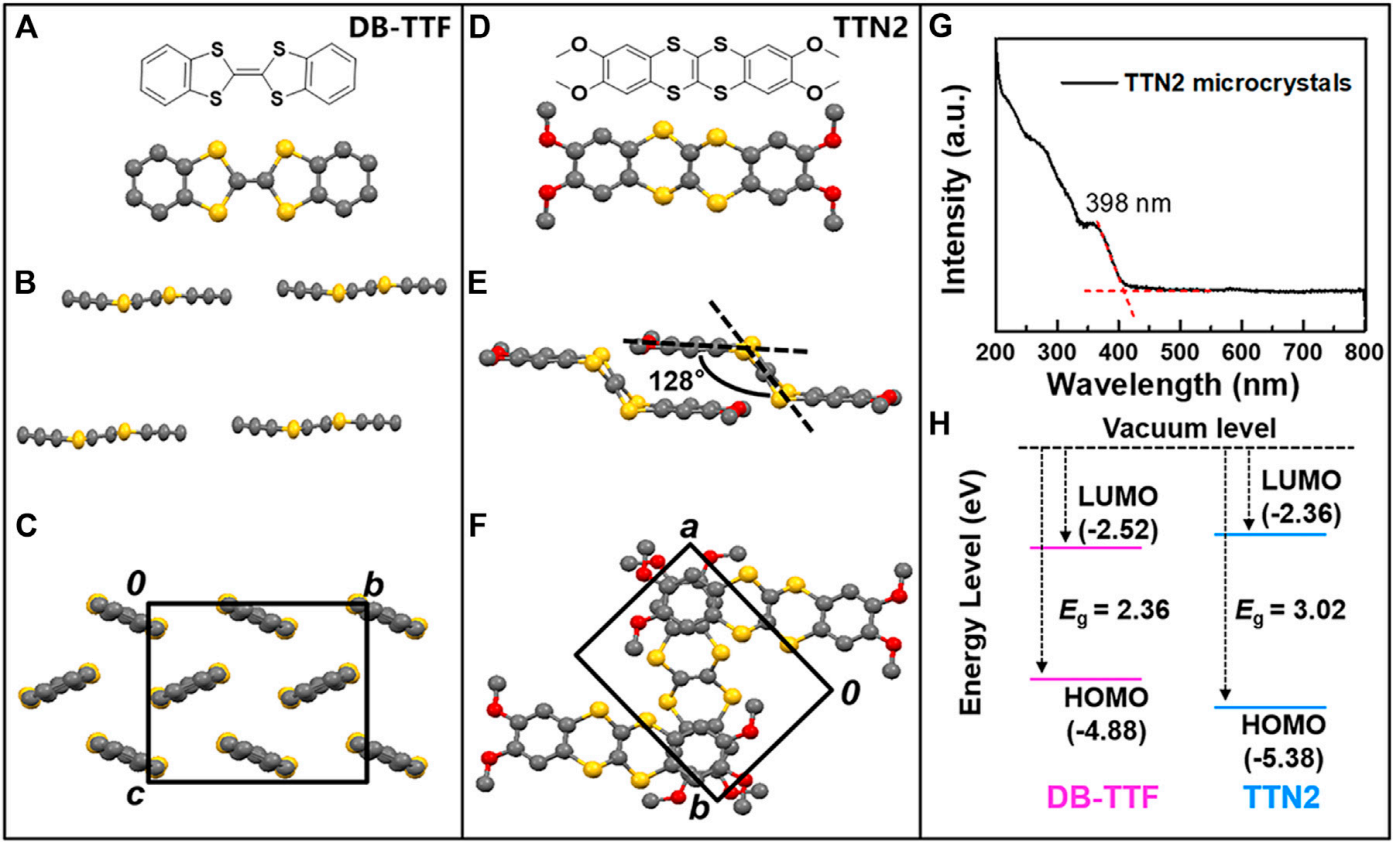
Molecular structure and packing motifs of **(A-C)** DB-TTF and **(D-F)** TTN2 **(G)** UV-Vis-NIR absorption spectra of TTN2 microcrystals **(H)** Energy levels and bandgaps of DB-TTF and TTN2.

Tetrathiafulvalene (TTF) has been extensively investigated as a strong electron donor in organic electronics (Canevet, et al., 2009; Wudl, et al., 1970). Among TTF derivatives, DB-TTF has attracted much attention (Figure 1A). It is a symmetrical, planar, and completely conjugated molecule, which resembles the prevailing structure of common OSCs (Wang, et al., 2018; Zhang, et al., 2018). DB-TTF shows a herringbone packing motif in the solids with a mean molecular plane distance of 3.60 Å (Figures 1A–C) (Emge, et al., 2007). It has been studied as a high performance OSC in OFETs (Mas-Torrent and Rovira, 2005, 2006b; Naraso, et al., 2005; Del Pozo, et al., 2016). However, in the solid state UV-Vis-NIR spectra, DB-TTF exhibits an absorption peak at 476 nm and the optical bandgap estimated from the absorption edge of the spectra was 2.36 eV (Mas-Torrent, et al., 2006a). The photoabsorption in VNIR range and the narrow optical bandgap indicate that OFETs based on DB-TTF are potentially unstable when irradiated by visible light and they are not suitable for applications that require visible-blind photoresponse (Mas-Torrent, et al., 2006a).

Tetrathianaphthalene (TTN) is an isomer of TTF which differs structurally from the latter only in the arrangement of the two ethyne bridges. In contrast to the planar molecular configuration of TTF, TTN adopts an armchair-like configuration with a dihedral angle between the terminal ethene and the central tetratia-enthene of about 137° (Varma, et al., 1988). It has been well-known that planarity of molecules plays a major role in determining the shape and wavelength position of the absorption spectra of materials (Nijegorodov and Downey, 1994). The decrease in planarity from TTF to TTN can cause blue shift of the absorption edge, making TTN a possible skeleton for stable OSCs. While aryl substituted or fused TTF derivatives have been intensively investigated in OFETs (Naraso, et al., 2005; Gao, et al., 2007; Yamashita, 2009; Mas-Torrent and Rovira, 2011, 2004; Yang, et al., 2013), the charge transport properties of TTN derivatives are still unknown. In this study, the optoelectronic properties of TTN2, a molecule with an armchair-like configuration, are uncovered. Figures 1D–F show the molecular structure and packing of TTN2, whose skeleton is an isomer of DB-TTF. As can be seen in Figure 1E, the dihedral angle between the central moiety (C_2_S_4_) and the terminal group in TTN2 was about 128° (Sun, et al., 2016). Compared with DB-TTF, TTN2 changed from a planar structure to an armchair configuration. UV-Vis-NIR absorption spectra of TTN2 solution and microcrystals (Figure 1G; Supplementary Figure S1) show that there is no absorption after 400 nm and the maximum wavelength absorption peak of TTN2 microcrystals is at 398 nm. Compared with DB-TTF with a maximum wavelength absorption peak at 476 nm (Mas-Torrent, et al., 2006a), the absorption of TTN2 shows a dramatical blue shift. Figure 1H shows the highest occupied molecular orbital (HOMO) and the lowest unoccupied molecular orbital (LUMO) levels of DB-TTF and TTN2. The HOMO levels of DB-TTF and TTN2 calculated by cyclic voltammetry (CV) are −4.88 eV and −5.38 eV, respectively (Mas-Torrent, et al., 2005; Sun, et al., 2016). The optical bandgaps of DB-TTF and TTN2 estimated from the solid state UV-Vis-NIR absorption spectrum are 2.36 eV (Mas-Torrent, et al., 2006a) and 3.02 eV, respectively. Therefore, the LUMO levels calculated from the HOMO levels and the optical bandgaps are −2.52 eV and −2.36 eV, respectively. We also obtained the frontier molecular orbitals (FMOs) and the HOMO and LUMO energy levels of DB-TTF, TTN2 and 1,4-benzodithiino[2,3-b][1,4]benzodithiine (TTN1) by theoretical calculations (B3LYP/6-31G (d,p)) based on their crystal structure (Supplementary Figure S2). The results showed that the increased optical bandgap of TTN2 was due to the change of its molecular configuration and the four methoxy groups showed little influence on the energy levels. By changing the molecular configuration from planar to armchair, the LUMO level becomes higher and the HOMO level becomes lower, indicating that controlling molecular configuration is an effective strategy to tune the bandgaps of OSCs.

**FIGURE 2:**
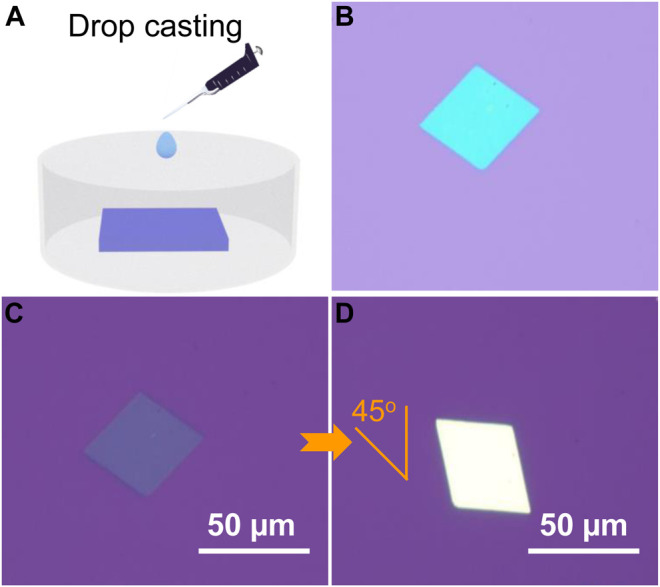
**(A)** Solution drop-casting to grow TTN2 microcrystals **(B)** OM and **(C, D)** POM images of TTN2 microcrystals.

**FIGURE 3:**
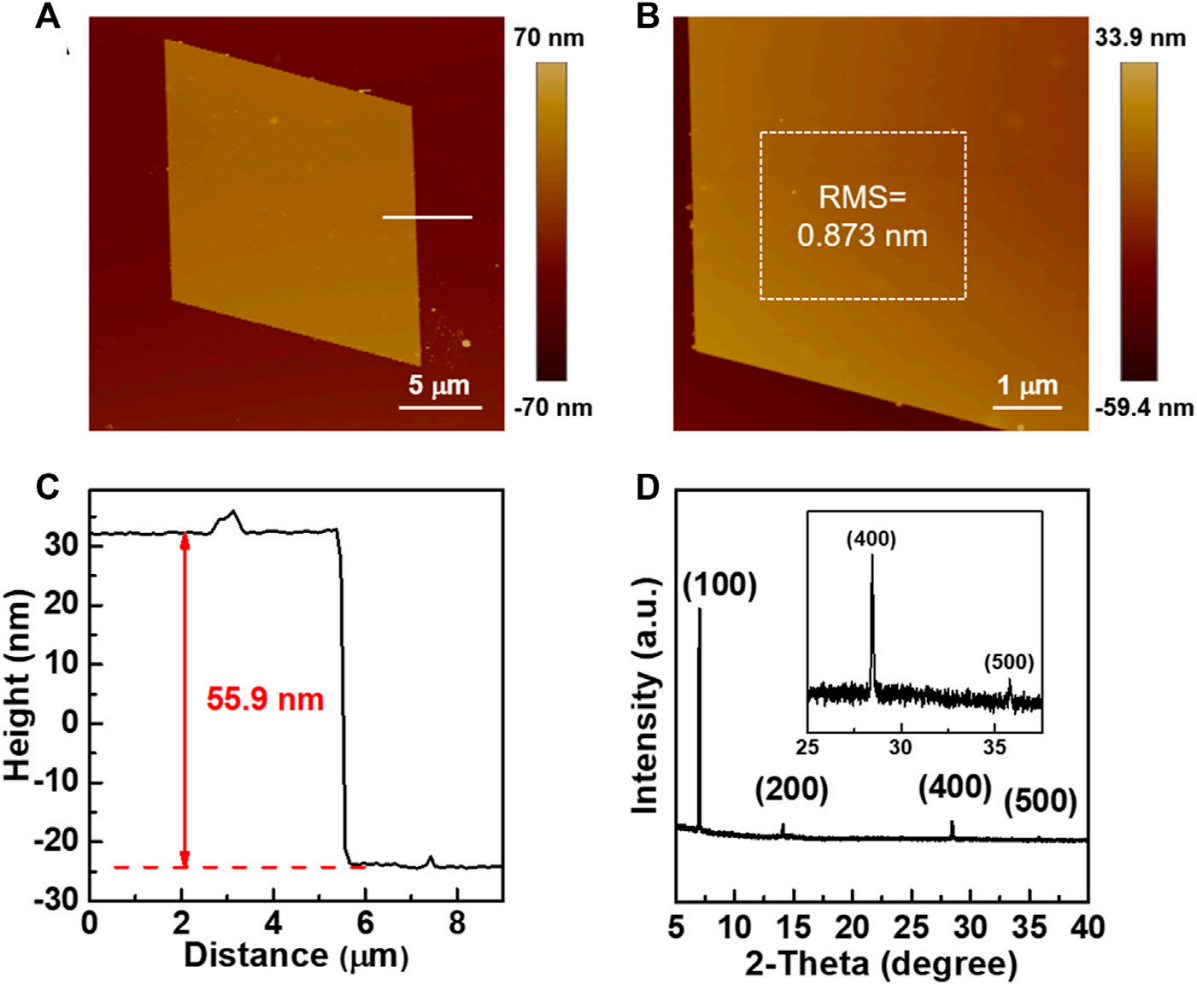
**(A, B)** AFM images of TTN2 microcrystals **(C)** Height profile of a TTN2 microcrystal **(D)** XRD spectra of TTN2 microcrystals.

In order to investigate the charge transport properties, TTN2 microcrystals were prepared by a simple solution drop-casting method (Figure 2A, see the Experimental method for details). Figure 2B shows an OM image of a TTN2 microcrystal, which exhibits a smooth surface without any notable cracks or steps. Under a POM, when the sample was rotated 45°, the color of the whole microcrystal changed uniformly and significantly from bright to dark (Figures 2C,D), indicating a crystalline nature of the microcrystal. The microcrystals were very stable—no morphology changes were observed by OM after the crystals being placed in ambient air and light for more than two years (Supplementary Figure S3). Figures 3A,B show typical AFM images of TTN2 microcrystals. The surface is atomically flat with a root-mean-square (RMS) roughness as low as 0.873 nm. Typical thickness of the as-grown TTN2 microcrystal is 55.9 nm (Figure 3C). The crystalline property of the TTN2 microcrystals was further assessed by out-of-plane XRD (Figure 3D). The smooth baseline and sharp diffraction peaks in the XRD spectra indicate that the TTN2 microcrystals are highly crystalline. The strong diffraction peak at 7.01°, which corresponds to a *d*-spacing of 12.6 Å, can be assigned to (100) plane. The corresponding second, fourth, and fifth order peaks at 14.1°, 28.4°, and 35.8° are also observed, indicating that (100) plane of the TTN2 microcrystals are parallel to the substrates. The sharp diffraction peaks in the XRD spectra of TTN2 microcrystals were unchanged after being placed in ambient air and light for more than two years, further indicating the high stability of the microcrystals (Supplementary Figure S4). Judging from the aforementioned results, TTN2 microcrystals possess high crystallinity and excellent stability.

**FIGURE 4:**
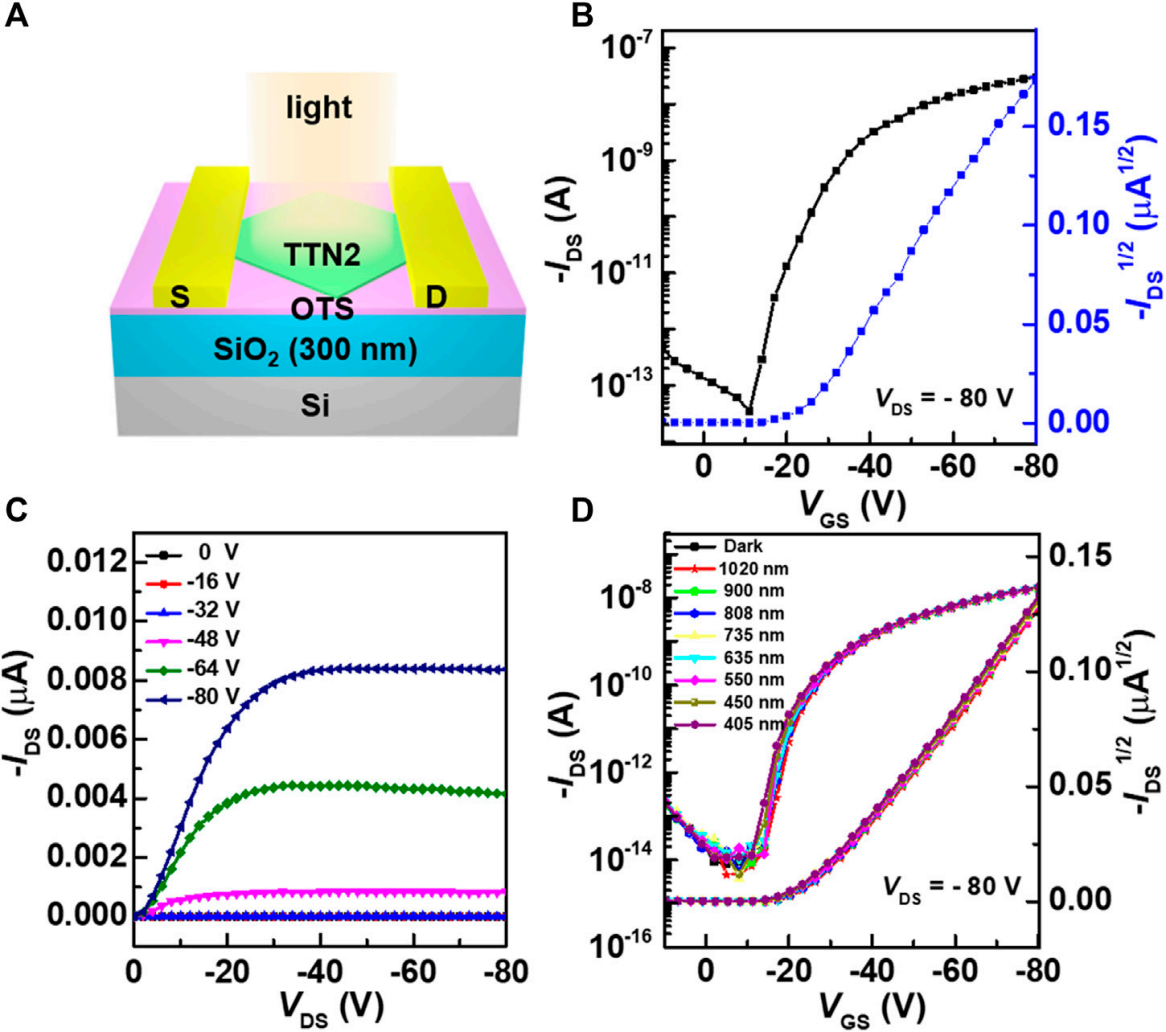
**(A)** Schematic diagram of a top-contact bottom-gate OFET **(B, C)** Typical transfer and output characteristic curves of the OFET. Channel length is 24 μm, Channel width is 45 μm **(D)** Photoresponse of the OFETs under laser irradiation of different wavelengths from 405–1,020 nm.

The charge transport properties and optoelectronic performances of TTN2 microcrystals were investigated by the construction OFETs with a bottom-gate top-contact configuration (Figure 4). The device structure is shown in Figure 4A. The transfer and output characteristic curves were tested in ambient air under dark conditions (Figures 4B,C). All devices showed typical p-type behaviors operating in accumulation mode. Judging from the dual sweep curves under dark conditions (Supplementary Figure S5), the devices exhibited a small hysteresis. High on/off ratio (*I*_on/off_) of approximately 10^7^ was obtained. The highest mobility reached 6.64 × 10^−2^ cm^2^ V^−1^ s^−1^ and the average mobility of 32 devices was 5.40 × 10^−3^ cm^2^ V^−1^ s^−1^ (Supplementary Figure S7A). Note that the results represent the first exploration of OFETs based on TTN derivatives. For the same device, after being placed in ambient air and light for more than two years, it still showed good field-effect performance (Supplementary Figure S6) and the average mobility of 32 devices could still reach 1.33 × 10^−3^ cm^2^ V^−1^ s^−1^ (Supplementary Figure S7B).

Judging from the UV-Vis-NIR absorption spectra and the optical bandgap of TTN2, OFETs based on TTN2 microcrystals are visible-blind. Figure 4D shows the photoresponse of the OFETs under laser irradiation of different wavelengths from 405–1,020 nm. No shift of the transfer characteristic curves was observed, indicating that both mobilities and threshold voltages of the OFETs were unaffected by VNIR light irradiation. In addition, under laser irradiation of different intensities, the curves also remained unchanged (Supplementary Figure S8). After that, we also tested the bias-stress stability of the devices. The transfer characteristic curves of the device showed negligible shifts during a continuous application of gate bias voltage for one hundred times (Supplementary Figure S9), which proved that the device had good bias-stress stability. Judging from the aforementioned results, OFETs based on TTN2 showed excellent environmental and operational stability.

## Conclusion

In summary, by controlling the molecular configuration from planar to armchair, a highly stable OSC TTN2 is obtained. Because of the large bandgap, TTN2 exhibits no photoabsorption in the VNIR region. OFETs based on TTN2 are insensitive to VNIR light and show excellent stability. The devices worked well after being stored in ambient air and light for over two years. Aryl-fused tetrathianaphthalenes with armchair-like configurations might be a new class of stable OSCs for highly stable OFETs.

## Data Availability

The original contributions presented in the study are included in the article/Supplementary Material, further inquiries can be directed to the corresponding authors.
