# Supplementary material for: Bandgap Engineering of an Aryl-Fused Tetrathianaphthalene for Visible-Blind Organic Field-Effect Transistors
Source: Front Chem. 2021 May 28;9:698246. doi: 10.3389/fchem.2021.698246 (PMC8193678; doi:10.3389/fchem.2021.698246)
Supplement: Supplementary file 1 [file DataSheet1.PDF]

Supplementary Material

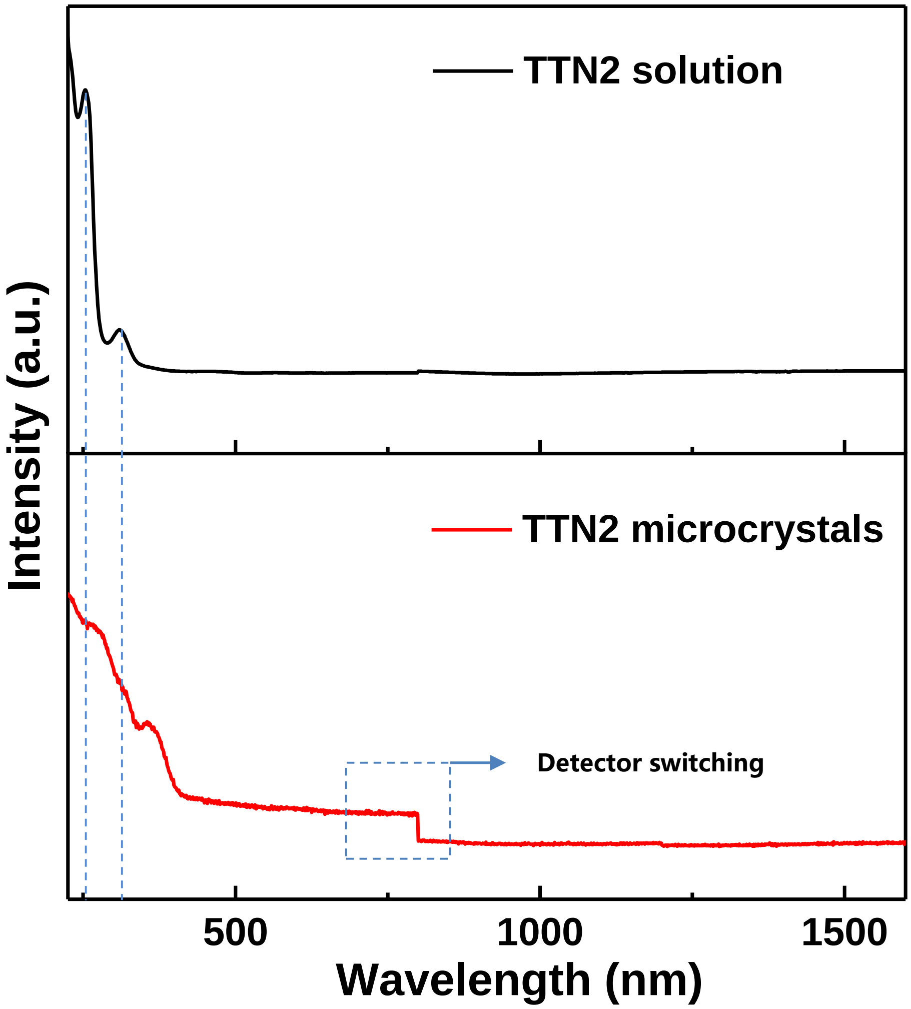

Supplementary Figure 1. UV-vis absorption spectrum of TTN2 in CH_2Cl_2 solution (c = 10^{-5} mol L^{-1}) and microcrystals.

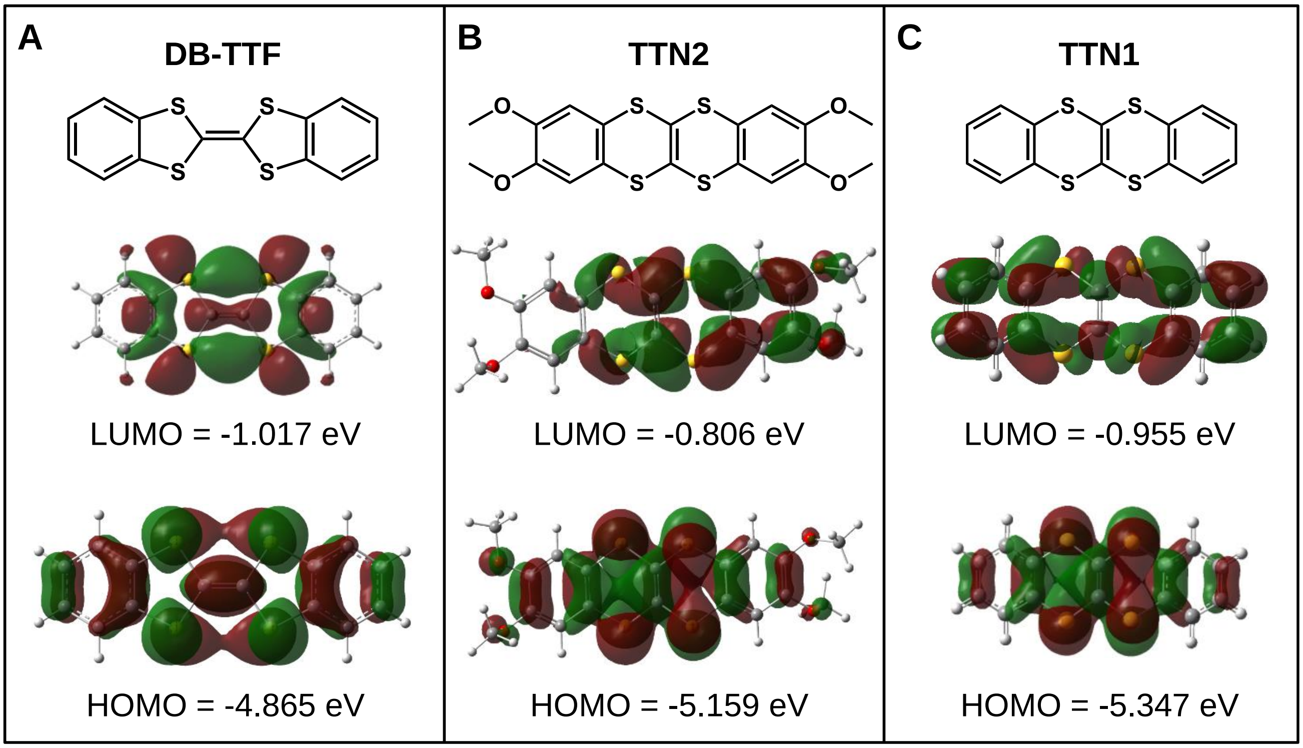

Supplementary Figure 2. The frontier molecular orbitals and the HOMO and LUMO energy levels of DB-TTF, TTN2 and TTN1 by means of the B3LYP/6-31G(d,p) level of theory.

By eliminating the four methoxy groups on TTN2, we obtained 1,4-benzodithiino[2,3-b][1,4]benzodithiine (TTN1). The optical bandgap of TTN1 calculated from its solid-state UV-Vis absorption spectrum is 2.97 eV (Sun, et al., 2016). The difference between the optical bandgaps of TTN2 and TTN1 is about 0.05 eV, so the effect of the four methoxy groups on optical bandgaps is very small and can be basically ignored. We also obtained the frontier molecular orbitals (FMOs) and the HOMO and LUMO energy levels of DB-TTF, TTN2 and TTN1 by theoretical calculations (B3LYP/6-31G(d,p)) based on their crystal structure (Supplementary Figure 2). The HOMO–LUMO energy gap (E_g = 3.848 eV) of DB-TTF is smaller than that of TTN2 (E_g = 4.353 eV). However, the HOMO–LUMO energy gap of TTN2 and TTN1 (E_g = 4.392 eV) are very similar. Therefore, according to the experimental results and theoretical calculations, the difference between the band gaps of TTN2 and TTN1 is very small, so the effect of methoxy groups on the energy level is very small and can be basically ignored.

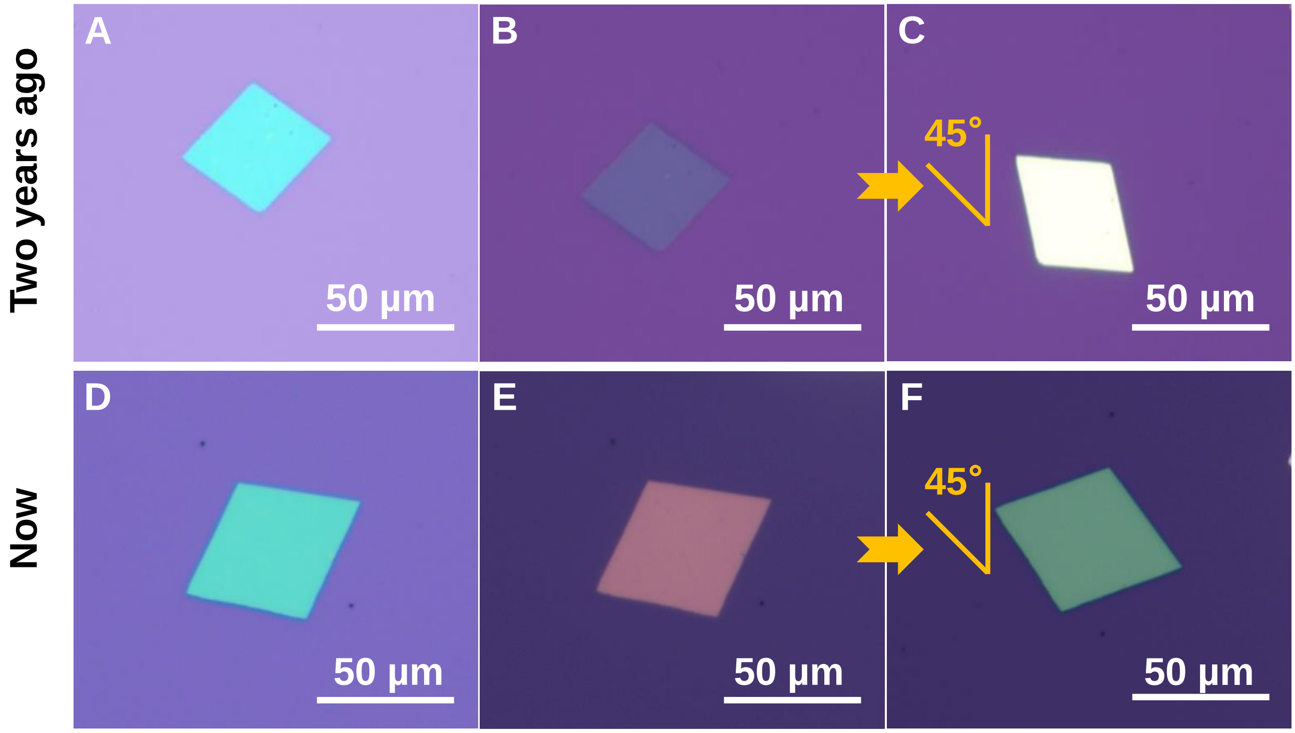

Supplementary Figure 3. (**A, D**) OM images and (**B, C and E, F**) POM images of TTN2 microcrystals before and after being placed in the air for two years.

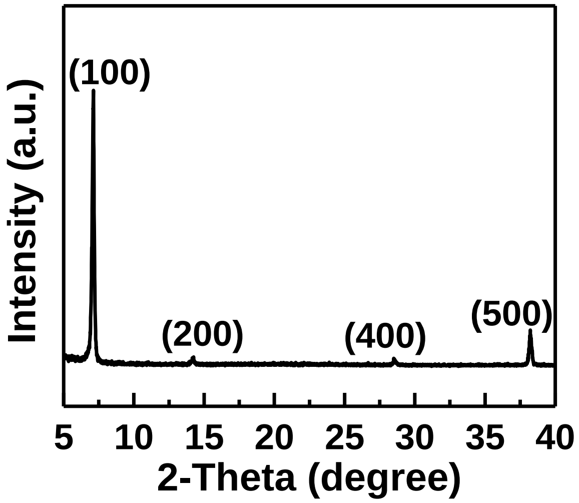

Supplementary Figure 4. XRD spectra of the TTN2 microcrystals after being placed in the air for two years.

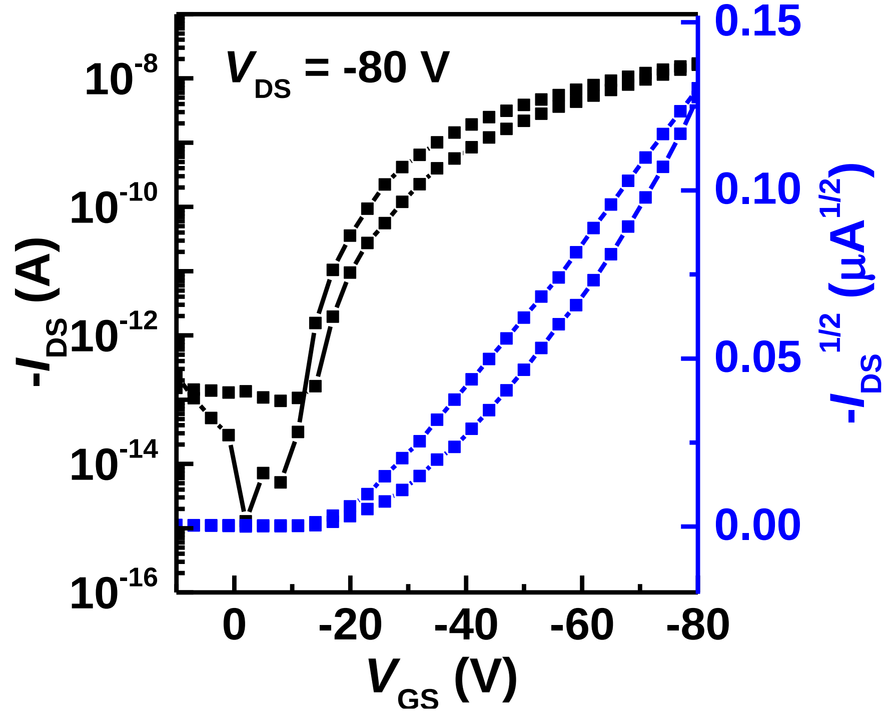

Supplementary Figure 5. Dual sweep for the transfer curves of the OFET based on TTN2 microcrystal in dark conditions.

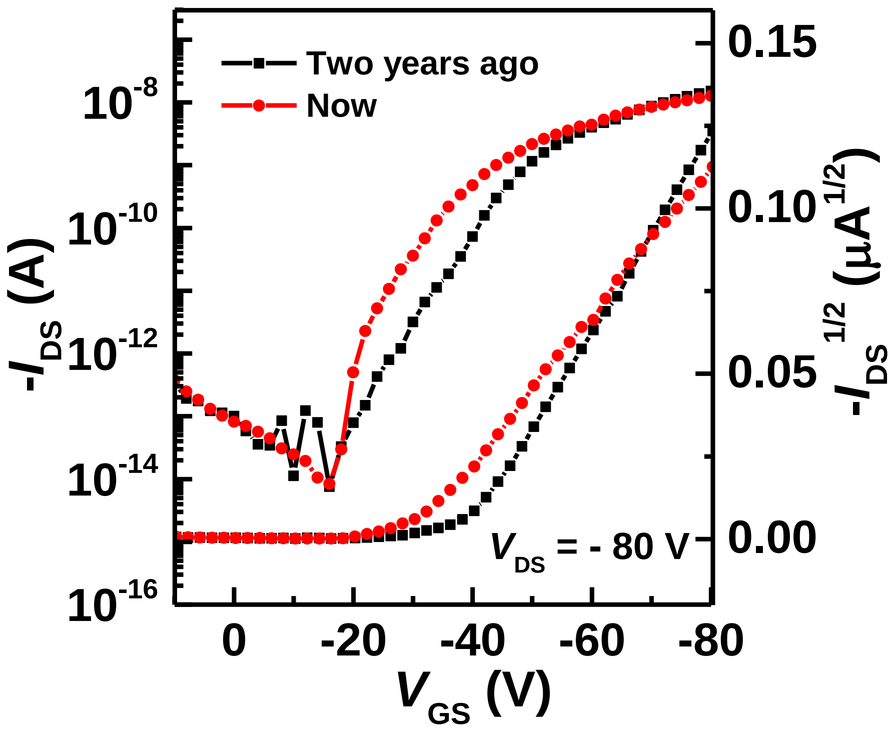

Supplementary Figure 6. Transfer curves of the OFETs based on TTN2 microcrystals before and after being placed in the air for two years.

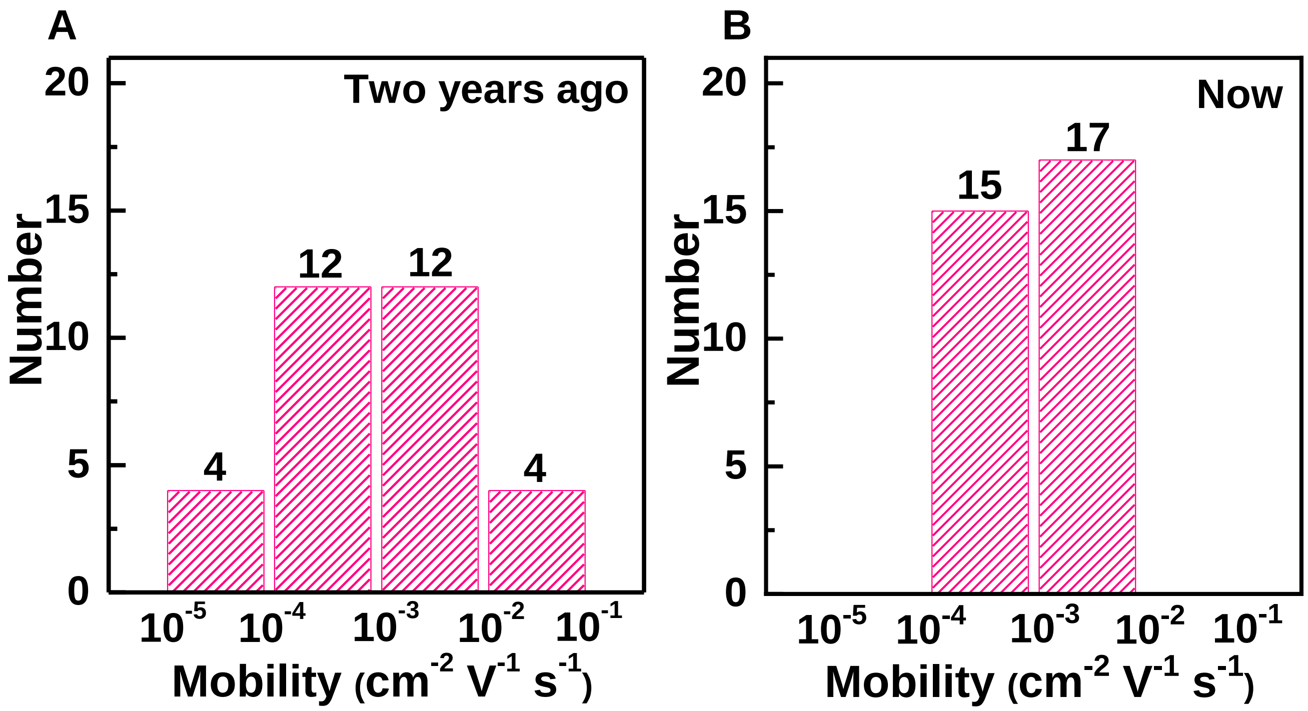

Supplementary Figure 7. Mobility distribution of 32 devices based on TTN2 microcrystals **(A)** before and **(B)** after being placed in the air for two years.

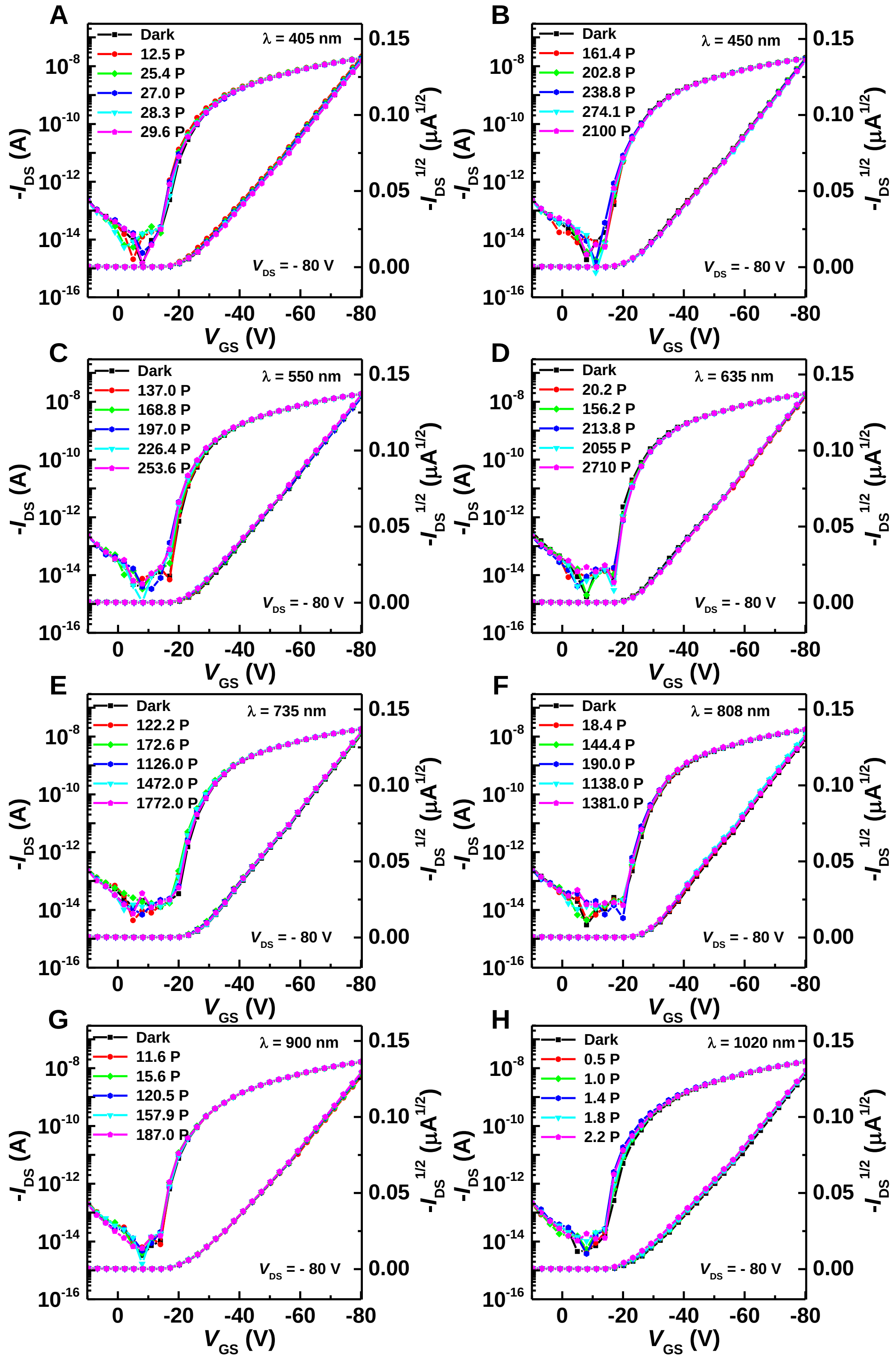

Supplementary Figure 8. OFETs based on TTN2 microcrystals measured in dark and under 400-1200 nm irradiation with varied intensities (1P = 1 μW cm^{-2}).

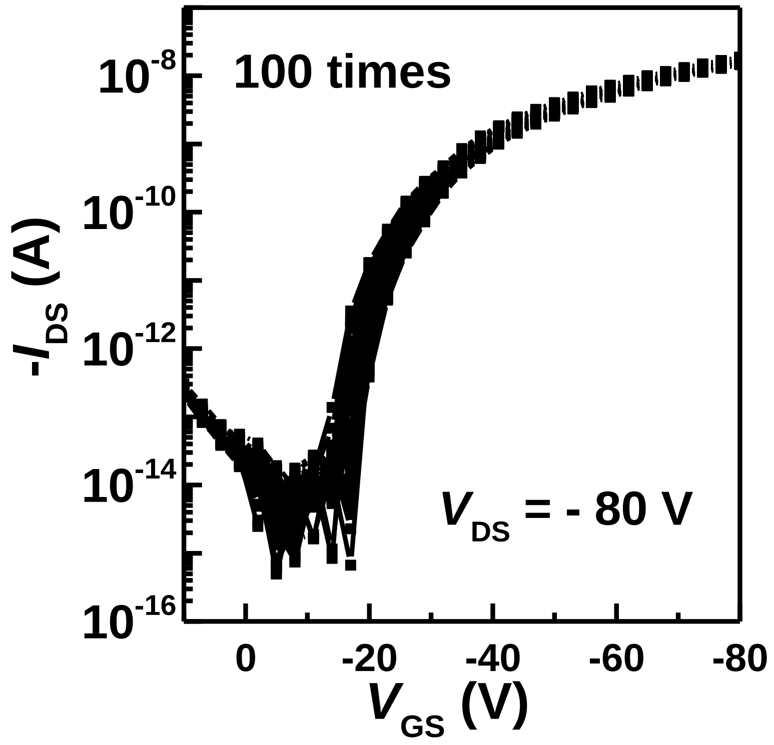

Supplementary Figure 9. Transfer characteristic curves of the device during the continuous application of bias voltage for one hundred times.

References

Sun, Y., Cui, Z., Chen, L., Lu, X., Wu, Y., Zhang, H.-L., et al. (2016). Aryl-fused tetrathianaphthalene (TTN): synthesis, structures, properties, and cocrystals with fullerenes. *RSC Adv.* 6, 79978-79986. doi: 10.1039/c6ra18945h
